# A retrospective chart review of clinical characteristics and magnetic resonance imaging findings of patients from a psychiatric facility in KwaZulu-Natal province, South Africa

**DOI:** 10.4102/sajpsychiatry.v25i0.1387

**Published:** 2019-11-27

**Authors:** Vidette M. Juby, Saeeda Paruk

**Affiliations:** 1 Discipline of Psychiatry, University of KwaZulu-Natal, Durban, South Africa

**Keywords:** structural neuroimaging, computerised tomography, magnetic resonance imaging, mental illness, Africa

## Abstract

**Background:**

Many neurological conditions manifest with psychiatric symptoms and may be misdiagnosed. Structural neuroimaging, that is, computerised tomography (CT) and magnetic resonance imaging (MRI), can aid in the diagnosis or exclusion of these conditions. Magnetic resonance imaging is preferable in this regard, but is more expensive and less readily available than CT. The indications for requesting MRI in the clinical psychiatric setting remain poorly defined. All published literature on the clinical utility of neuroimaging in Africa is on CT scans.

**Aim:**

The aim of this study was to describe the clinical characteristics and MRI findings in a cohort of patients presenting with psychiatric symptoms.

**Setting:**

A specialist psychiatric training hospital, Townhill Hospital, in Pietermaritzburg, KwaZulu-Natal.

**Methods:**

A retrospective chart review of all patients who underwent MRI between 01 October 2010 and 31 June 2016 was done. Magnetic resonance imaging findings were correlated with socio-demographic and clinical information, including psychiatric diagnosis, indication for MRI imaging and effect on clinical management.

**Results:**

Fifty-three MRIs were performed. Thirty-three (62%) were abnormal. Patients with HIV, neurocognitive disorders, chronic mental illness and involuntary admission were more likely to have abnormal scans (83%, *p* = 0.089; 80%, *p* = 0.496; 71%, *p* = 0.089 and 79%, *p* = 0.021, respectively). The findings of 54% of abnormal MRIs (24% of all MRIs performed) resulted in referral to other disciplines. No statistically significant associations were found with socio-demographic or clinical factors.

**Conclusion:**

Abnormalities on MRI scans in mentally ill patients were common and a quarter of patients required referral to other disciplines. Further studies are required to clarify the clinical utility of MRI in patients with psychiatric illness, which could assist in the development of a guide for the rational use of this modality in a resource-constrained environment.

## Introduction

Certain brain lesions and neurological conditions can manifest with psychiatric symptoms.^1,2,3,4^ For example, brain tumours may present with mood, memory and/or psychotic symptoms.^4^ Furthermore, psychiatric conditions are frequently comorbid in the context of organic brain disease, such as depression in traumatic brain injury and stroke, cognitive impairment in vascular disorders, and psychotic symptoms in Parkinson’s disease. Up to half of patients with epilepsy will have psychiatric symptoms.^5^ These neurological conditions need to be excluded from primary psychiatric illnesses as they require different and, at times, definitive management.

Neuroimaging in the form of structural computed tomography (CT) and magnetic resonance imaging (MRI) can aid in the diagnosis or exclusion of underlying neurological conditions. Consensus is that CT may be preferable for suspected intracranial and subdural bleeding, skull fracture or in patients with pacemakers or other contraindications to MRI. Magnetic resonance imaging provides much greater contrast between brain structures, and thus better soft tissue detail. However, CT is much more readily available and relatively cheaper than MRI.^6,7^

Wahlund et al. retrospectively studied 731 MRI scans of patients with psychiatric symptoms and 121 (17%) had abnormal scans.^8^ They conclude that MRI examination is valuable in the investigation of psychiatric symptoms in that it aids in ruling out organic causes. This sentiment is shared by Erhart et al. who reviewed the results of 253 psychiatric patients who underwent MRI. In their study, in 38 (15%) cases, treatment was modified.^9^

In the resource-limited setting of South Africa or sub-Saharan Africa, there is limited data on the clinical utility of MRI in mental health patients. Furthermore, there are currently no clear guidelines in terms of the indication for MRI neuroimaging in psychiatric patients. All published literature focuses on CT findings.^10,11,12,13,14^ This study aimed to describe the prevalence and spectrum of abnormalities found on MRI scans in patients presenting with psychiatric symptoms. A secondary aim was to assess the impact of MRI outcomes on subsequent diagnostic processes and/or clinical management.

## Research methods and design

A retrospective chart review of all patients from Townhill Hospital who underwent MRI scans between 01 October 2010 and 31 June 2016 was done. Townhill Hospital is a specialist psychiatric training hospital in Pietermaritzburg, KwaZulu-Natal, with in- and outpatient services. Where any data were missing for analysis, this was reported, and the respective patient was excluded from analysis.

Magnetic resonance imaging scans were requested at the discretion and clinical judgement of the treating consultant psychiatrist when the presence of organic pathology was deemed likely. Magnetic resonance imaging scans are frequently requested when a CT scan is abnormal, but may also be requested as a first-line neuroimaging if the suspected condition would not be adequately imaged by CT.

Cases were identified by examining the MRI daily work register at Greys Hospital that records the patient name, date of birth and referring hospital. The MRI request form and reports of these scans were reviewed as well as any other imaging performed, specifically CT. If the CT scan had been performed in the 12 months prior to the MRI at the same facility, this report was also reviewed and the results recorded. The MRI scans were performed using a Philips 1.5 Tesla MRI scanner and were reported on by radiology registrars and checked and signed off by the consultant radiologist on duty at the time.

The clinical hospital files were then reviewed at the psychiatric hospital that allowed the extraction of demographic information (gender, age, race, residential category, highest level of education) and clinical data (HIV status, clinical illness course, psychiatric diagnosis and indication for neuroimaging). The psychiatric diagnosis of patients scanned before 2013 when DSM IV-TR was in use were converted to the corresponding DSM-5 diagnosis for uniformity.^15^

The results of the MRI scans were correlated with the demographic and clinical data collected from the patient psychiatric files. Results of CT scans performed within 12 months of the MRI were also recorded. The impact of the MRI findings on clinical management was recorded as diagnosis change, confirmation of diagnosis, change in management, no change in management and referral to other discipline. All the data were collected by the first author (V.M.J.) using a structured data sheet.

Data were analysed using Statistical Package for the Social Sciences (SPSS), version 25. Descriptive statistics were calculated for demographic variables, clinical criteria and neuroimaging findings. Categorical data were compared using a chi-squared test or Fischer’s test as deemed appropriate. The authors set alpha at 0.05, and a significance level of *p* < 0.05 was used for statistical significance testing.

### Ethical considerations

The required ethical approval was obtained from the relevant Biomedical Research Ethics Committee and the Department of Health.

## Results

During the study period of over 5 years, 53 patients underwent MRI. Clinical data for two patients were incomplete for admission status (i.e. involuntary, voluntary or assisted admission) at booking and one patient for the working psychiatric diagnosis and the clinical course of the mental condition. These patients were excluded from the relevant analyses, but included for all other analyses.

The mean age of patients who were scanned was 34.43 years (s.d. ± 15.3). There were 29 male and 24 female patients. Socio-demographic and clinical characteristics of patients who underwent MR neuroimaging are reported in Table 1. Most scans were performed on inpatients (*n* = 32; 60%), the majority of whom were involuntary mental healthcare users (*n* = 24; 45%) at the time and presenting with more than one episode of mental illness (*n* = 35; 66%). Scans in chronic or relapsing patients were more likely to be abnormal than those performed on patients presenting with their first episode of psychiatric illness (71% vs. 47%, *p* = 0.089).

**TABLE 1:** Socio-demographic and clinical characteristics of patients according to magnetic resonance imaging scan result.

Socio-demographics and clinical characteristics	Total		Abnormal		Normal		*p*
	*n* = 53	%	*n* = 33	%	*n* = 20	%	
**Gender**							
Male	29	55	21	64	8	28	
Female	24	45	12	50	12	50	0.154
**Race**							
Black	34	64	20	59	14	41	
White	8	15	6	75	2	25	
Indian	8	15	5	63	3	38	
Mixed race	3	6	2	67	1	33	0.957
**Residential**							
Rural	14	26	9	64	5	36	
Urban	36	68	22	61	14	39	1.00
**Admission status at booking**							
Inpatient	45	85	27	60	18	40	
Outpatient	6	11	5	83	1	17	
Missing	2	4	1	50	1	50	0.472
Voluntary	13	25	8	62	5	39	
Assisted	7	13	1	14	6	86	
Involuntary	31	59	23	74	8	25	0.017
**Admission status at scan**							
Inpatient	32	60	22	69	10	53	
Outpatient	19	36	10	31	9	47	0.464
Missing	2	4	1	50	1	50	
Voluntary	21	40	12	57	9	43	
Assisted	6	11	1	17	5	83	
Involuntary	24	45	19	79	5	21	0.021
**Clinical course**							
First episode	17	32	8	47	9	53	
Recurrent episodes	35	66	25	71	10	29	
Missing data	1	2	1	100	0	0	0.089
**HIV status**							
Positive	6	11	5	83	1	17	
Negative	35	66	23	66	12	34	
Unknown	12	23	5	42	7	58	0.089
**Working diagnosis**							
Psychosis	25	47	15	60	10	40	
Mood or anxiety disorder	18	34	12	67	6	33	
Neurocognitive disorder	5	9	4	80	1	20	
Other	4	8	1	25	3	75	
Missing data	1	2	1	100	0	0	0.496

### Magnetic resonance imaging results

Of the 53 scans performed during the study period, 33 (62%) were reported to be abnormal. Abnormal findings varied greatly, but included age-inappropriate atrophy (generalised or localised) (*n* = 18), cysts (*n* = 4), tumours (*n* = 3), periventricular leukoencephalopathy (*n* = 3), pituitary nodule and hyperplasia (*n* = 2), post-traumatic gliosis (*n* = 2), arterio-venous malformations (*n* = 2), loss of volume of caudate nuclei (*n* = 2) and subcortical small vessel disease (*n* = 2).

### Clinical profile

Six (11%) of the 53 patients were HIV-infected, 35 were HIV-negative and HIV status was unknown for 12 (23%) of the patients. CD4 count for infected individuals varied between 73 cells/mL and 1179 cells/mL with a mean of 500 (SD ± 410.6) cells/mL. Five out of six (83%) of MRIs on HIV-infected patients were abnormal.

The most common working diagnosis of patients who underwent MRI was psychosis (*n* = 25; 47%) followed by mood or anxiety disorders (*n* = 18; 34%). Five patients (9%) were assessed as having major neurocognitive disorders and four (8%) had other psychiatric diagnoses, namely two patients with presumed conversion disorder, one with unspecified disruptive disorder and one with Tourette’s syndrome. Data were missing for one patient. Four out of five (80%) patients with neurocognitive disorders had abnormal MRI scans.

The most common clinical indications for MRI were abnormal CT scan result (*n* = 25; 47%), delirium or cognitive impairment (*n* = 12; 23%), personality or behaviour change (*n* = 12; 23%), abnormal laboratory result (*n* = 11; 21%), history of head injury (*n* = 10; 19%) and history of seizures (*n* = 7; 13%). Table 2 summarises clinical indications for neuroimaging.

**TABLE 2:** Clinical indication for patients with normal and abnormal scans.

Clinical Indication	Total		Abnormal		Normal		*p*
	*n* = 53	%	*n* = 33	%	*n* = 20	%	
Screening only	1	2	1	100	0	0	1.00
Cognitive impairment or delirium	12	34	9	75	2	25	0.50
Personality or behaviour change	12	34	8	67	4	33	1.00
Focal neurological signs	5	9	3	60	2	40	1.00
Abnormal EEG	2	4	0	0	2	100	0.14
Abnormal test result	11	21	6	55	5	46	0.73
Previous CT result	25	47	17	68	8	32	0.57
Patient complaint	1	2	0	0	1	100	0.52
Physical sign	5	9	2	40	3	60	0.35
Previous history of event or illness	2	4	1	50	1	50	1.00
History of seizures	7	13	5	71	2	29	0.70
History of head injury	10	19	7	70	3	30	0.73
Suspected dementia	3	6	3	100	0	0	0.28
Alcohol or substance abuse	6	11	4	67	2	33	1.00
History of known organic disease	1	2	1	100	0	0	1.00
Other	3	6	3	100	0	-	0.58

CT, computerised tomography; EEG, electroencephalogram.

### Computerised tomography and magnetic resonance imaging outcome

Comparison of CT and MRI findings was only carried out if the CT scan was performed at the same general hospital within 12 months prior to the MRI. These criteria were met in 23 patients (43%) of the 53 patients with MRI and 16 (70%) of the CT scans were reported as being abnormal. Of those with abnormal CT scans, 13 (81%) had abnormal MRI scans also. Three patients with abnormal CT scans had normal MRI scans and three patients with normal CT scans had abnormal MRI scans (see Table 3).

**TABLE 3:** Magnetic resonance imaging results in relation to computerised tomography results.

CT Results	Total		NormalMRI		Abnormal MRI		*p*
	*n* = 23	%	*n* = 16	%	*n* = 7	%	
Normal CT	7	30	4	57	3	43	-
Abnormal CT	16	70	3	19	13	81	0.091

CT, computerised tomography; MRI, magnetic resonance imaging.

### Magnetic resonance imaging and clinical outcome

The clinical outcome of patients who underwent MRI is reported in Table 4. Referral to other clinical disciplines (internal medicine, neurology and neurosurgery) was indicated in 13 patients (54% of those with abnormalities on MRI). The management of 35 (66%) of the patients was unchanged after MRI.

**TABLE 4:** Clinical outcome according to magnetic resonance imaging result.

MRI result	Diagnosis change	Confirm diagnosis	Change in management	No change	Referral to other discipline
**Normal**					
*n* = 20	0	0	0	20	0
%	0	0	0	100	0
**Abnormal**					
*n* = 33	1	3	1	15	13
%	3	9	3	46	39
**Total**					
*n* = 53	1	3	1	35	13
%	2	6	2	66	25

MRI, magnetic resonance imaging.

## Discussion

In the current study, 62% of MRI scans were reported to be abnormal. This is a much higher rate than the findings of other studies where the incidence of abnormalities varied from 17% to 55%, though direct comparison to these studies is not possible because of differences in methodology.^8,9,16,17^ For example, Wahlund and colleagues excluded patients with neurological signs and symptoms and their study population was mostly patients in their first episode of mental illness, and Erhart and colleagues excluded patients who were suspected to have dementia.^8,9^ To the authors’ best knowledge, there is no literature on MRI scans of patients with psychiatric illness from Africa. The higher rate of abnormal findings in this study may be because of more stringent selection criteria by local clinicians as MRI access is limited because of health resource constraints.

This study aimed to describe the prevalence and spectrum of abnormalities found on MRI scans in patients presenting with psychiatric symptoms. During the study period of approximately 5 years, 53 MRIs were performed for a 280-bed psychiatric hospital. Available admission registers (from 2013 to 2016) show an average of 721 inpatient admissions per year. The outpatient department sees an average of 6826 patients per year of which 582 are new patients. It is thus evident that MRIs are very selectively requested for patients at the psychiatric unit. Abnormal MRI results prompted elective referral to another discipline in a quarter (25%) of cases.

Most patients who underwent MRI were involuntary inpatients at the time of booking (*n* = 31, 59%) and were seldom discharged prior to scanning; 24 patients were still involuntary inpatients at the time of scan. This could reflect that the increased severity of, or refractory nature of, their symptoms was an indication for neuroimaging. There was a significant association between admission status and MRI outcome, with patients who were assisted mental healthcare users in terms of the South African Mental Healthcare Act less likely to have an abnormal scan result (five out of six normal – 83%, *p* = 0.021) than involuntary mental healthcare users. These findings suggest that increased disease severity suggested by admission status may prompt neuroimaging and may be an indicator of underlying structural neurological anomalies.^18^

Fewer scans were performed for mental healthcare users in their first episode of mental illness than those who were presenting with recurrent mental illness (66% vs. 33%), with the yield of abnormal MRIs being lower than those with recurrent episodes (47% vs. 74%). This finding is in keeping with the findings of Goulet et al. who, after analysing 184 MRIs in patients with first episode psychosis (FEP), concluded that ‘structural brain imaging is unlikely to show anomalies in first episode psychosis in otherwise healthy people’.^19^ In contrast, a study by Adams et al. found that 62.5% of 112 MRIs performed on patients with FEP had incidental findings, but only three patients (2.7%) had lesions that could have been potential causes of psychosis.^20^ It is worth noting that the mean age of patients in their study was 59.3 years, which could account for the higher incidence of incidental findings.

The HIV prevalence rate in the current study of 11.3% is substantially lower than the HIV prevalence rate in mental healthcare users in other South African studies that range from 23.8% to 44%.^21,22,23^ It is of concern that the HIV status of 22.6% of the patients was unknown. This may be because of challenges with obtaining informed consent with some mental healthcare users, but may also be because of an inadequate HIV screening policy. The need for HIV screening for all patients with severe mental illness needs to be reinforced, as it may have implications on treatment and outcome. Five (83%) of the six HIV-infected subjects had abnormal MRIs of which four (67%) required referral to another discipline amounting to 31% of all the referrals. These results suggest that HIV-infected individuals are more likely to have clinically significant abnormalities on MRI scan and HIV status may be a useful indicator for MRI. This finding is consistent with the findings of a study of 305 MRIs performed on HIV-positive individuals over a 15-year period, which reported 263 (86%) had abnormal scans, the majority being subcortical white matter changes followed by cerebral atrophy.^24^ Thus, the utility of MRI scanning in HIV-infected individuals with psychiatric symptoms in resource limited settings requires further exploration to facilitate the development of clinical guidelines.

The working diagnosis of the patients who underwent MRI was largely in keeping with other similar studies of neuroimaging in psychiatry.^16,17^ When the MRI was carried out for patients with suspected neurocognitive disorder, there was a high rate of abnormal findings. However, only two of the five patients (40%) required further referral. These two patients were also HIV-positive.

Almost half (47%) of the patients who had MRI had an abnormal CT scan as an indication for MRI scan reflecting the tendency to use CT first line as it is more accessible and serves as a so-called ‘screening test’. This finding may be because of, in the current study setting, CT scans being more readily available and with shorter waiting times. This was not reflected as an indication in any of the other literature reviewed. Other indications for MRIs seemed to be under-represented in this study, for example focal neurological signs and abnormal EEGs. In this study, these were indications for MRI in 9% and 4% of patients, respectively, which is significantly less than the 21.3% and 26.8% reported by Mueller et al.^17^ This may reflect the tendency to refer patients with neurological signs and symptoms to the neurologists for further assessment and investigation prior to neuroimaging. This may also be because of site bias as patients with focal neurological signs and seizures are managed in general hospitals and not psychiatric hospitals.

Magnetic resonance imaging findings very seldom resulted in a change of psychiatric diagnosis, but did result in referral to another discipline for further management implying the possibility that the psychiatric symptoms could be as a result of underlying neurological disease. The majority of patient’s psychiatric management remained unchanged despite abnormal MRI findings. Although 13 patients required referral to another discipline, no referrals were urgent. This is similar to the findings in other studies: Mueller et al. reported a change of diagnosis in only 0.5% of patients, and urgent referral of 4.8% of patients.^17^ A review of four MRI studies in psychosis found that approximately 5% of patients had clinically significant findings on MRI, which would result in any change to management.^25^

Recommendations for MRI imaging in patients presenting with psychiatric symptoms need to be formulated. Findings from this study suggest MRI scans may be of clinical significance to exclude neurological pathology and MRI must be considered in selected patients such as those with refractory or severe symptoms not responding to treatment, abnormal CT neuroimaging, HIV infection and suspected neurocognitive disorder, which all tended to yield more abnormal MRI outcomes.

This study is limited by several factors. Firstly, it was limited by the small number of MRI scans performed, thus limiting the power of the findings. Furthermore, no neuroimaging protocol is in place, thus MRIs are requested at the discretion of the treating psychiatrist and performed at the discretion of the radiologist. Often when an organic cause for psychiatric symptoms is suspected, the psychiatric patient may be co-managed with the department of neurology. Magnetic resonance imaging scans are more easily obtainable by the neurology team and those patients would not have been included in this study. A retrospective chart review is dependent on the quality of the record keeping and clinical notes, and this may limit study findings. The study was based at one urban, tertiary level psychiatric hospital, and thus results may not be generalisable to patients presenting at the general hospital level with psychiatric symptoms.

## Conclusion

The clinical and diagnostic indications for scans in this study are in keeping with international studies, but our study demonstrated a higher rate of MRI abnormality and referral to other disciplines. This may be because of the context of more stringent clinician discretion when requesting MR neuroimaging being mindful of healthcare costs and resource limitations. The study findings suggest that MRI is useful to exclude an organic cause in clinically indicated groups of patients with psychiatric illness, in particular those with neurological signs and those with HIV. There is a need for further study of the utility of MRI in patients admitted to psychiatric units and for the development of local guidelines on the clinical indications for MRI in patients presenting with psychiatric symptoms.
